# Modelling C9orf72 dipeptide repeat proteins of a physiologically relevant size

**DOI:** 10.1093/hmg/ddw327

**Published:** 2016-10-23

**Authors:** Janis Bennion Callister, Sarah Ryan, Joan Sim, Sara Rollinson, Stuart M. Pickering-Brown

**Affiliations:** Division of Neuroscience and Experimental Psychology, Faculty of Biology, Medicine and Health, University of Manchester, AV Hill Building, Oxford Road, Manchester, UK

## Abstract

C9orf72 expansions are the most common genetic cause of FTLD and MND identified to date. Although being intronic, the expansion is translated into five different dipeptide repeat proteins (DPRs) that accumulate within patients’ neurons. Attempts have been made to model DPRs in cell and animals. However, the majority of these use DPRs repeat numbers much shorter than those observed in patients. To address this we have generated a selection of DPR expression constructs with repeat numbers in excess of 1000 repeats, matching what is seen in patients. Small and larger DPRs produce inclusions with similar morphology but different cellular effects. We demonstrate a length dependent effect using electrophysiology with a phenotype only occurring with the longest DPRs. These data highlight the importance of using physiologically relevant repeat numbers when modelling DPRs.

## Introduction

C9orf72 expansions are the most common genetic cause of frontotemporal lobar degeneration (FTLD) and motor neuron disease (MND) identified to date and are found in approximately one out of every twelve patients diagnosed with these diseases (1–3). The protein product of this gene is of unknown function, however, *in silico* analysis suggests it may be related to DENN ‘differentially expressed in normal and neoplastic cells’ GDP exchange factors (GEFs) proteins (4,5). Patients with this expansion also have a particular hippocampal and cerebellar pathology that is not seen in other types of FTLD and MND, which results from the unconventional translation of the expanded repeat on both strands producing 5 separate dipeptide repeat proteins (DPRs) of unknown significance (6–8). The molecular mechanisms through which the expansion leads to neurodegeneration are currently unclear, however, it is likely to result from one, or a combination, of three possible mechanisms. These are 1) haploinsufficiency (there are multiple papers in the literature reporting a reduction of C9orf72 in patients (2,3,9,10)), 2) toxic RNA nuclear foci that likely sequester important proteins (such as Pur Alpha or hnRNP A3 and H amongst others (11–13), or 3) aggregating potentially toxic DPRs that are translated from the repeat (7,8). A recent study reported that DPR pathology did not correlate with neurodegeneration in patient tissue (14), however, it is possible that soluble forms of the peptides are toxic, before formation of insoluble aggregates. Several studies have reported that it is the DPRs not the RNA foci that are responsible for toxicity at least in Drosophila models and cell culture (15–17).

Animal and cellular models of the DPRs are emerging but these are not perfect because they do not express DPRs of a size similar to those observed in patients (16–18). Importantly, it has already been demonstrated that DPR length has an effect, with longer repeats having greater toxicity (19) and the ability to impair nucleocytoplasmic transport (20). Recently, there has been a report of an individual with 30 hexanucleotide repeats in C9orf72 that died without any clinical signs of cognitive issues or motor impairments (21). In addition, they had no signs of neurodegeneration but did have DPR pathology. A second case-study described an individual with 70 repeats and no clinical symptoms (22). Therefore, it appears that short repeats in animal and cellular models can induce neurodegeneration but do not appear to in humans. This suggests that the physiological mechanisms occurring in models with short repeats could be different to those involved in human disease. Therefore, to better explore and understand the physiological mechanisms that are affected by DPR presence, it is vital to generate models with DPRs of a length observed in human patients. To address this, we have now generated expression constructs that encode DPRs using alternative codons to reduce the repetitiveness of the DNA sequence, increasing their stability in bacteria and allowing DPR expression in the absence of G_4_C_2_ RNA. This has allowed us to clone DPRs in excess of 1000 repeats, matching DPR size observed in human disease. These constructs when expressed in cells recapitulate many features observed in human pathology. In addition, we demonstrate, using electrophysiology, that different sized DPRs have different physiological effects with the longest having the most profound. These findings suggest that models expressing smaller DPRs could produce data that is of questionable relevance and that DPRs of pathophysiological length should be used when modelling C9orf72 related disease.

## Results

### DPR expression

**Figure 1.**
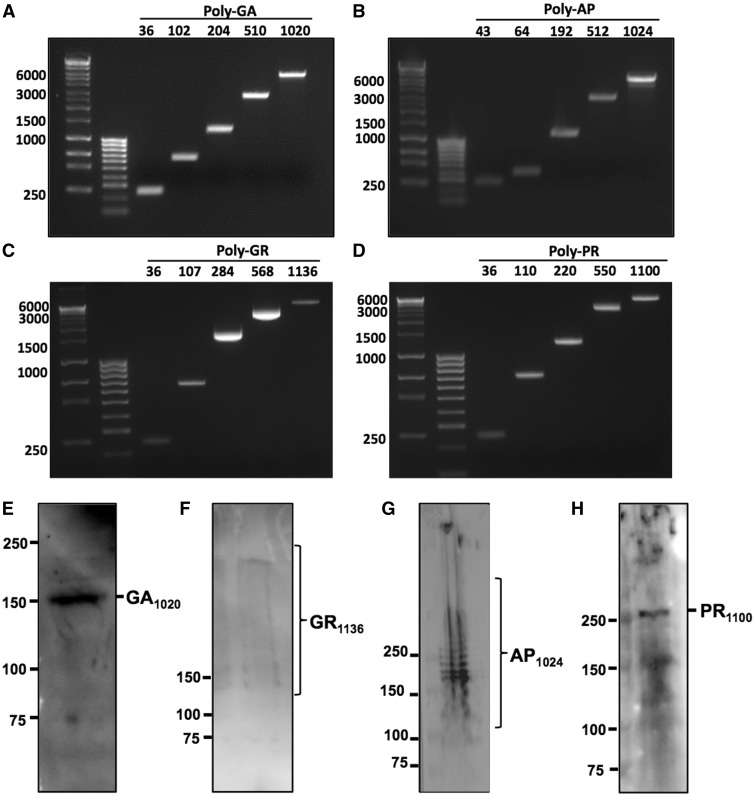
Ethidium bromide-labelled agarose gels showing repeat sequences from GFP-tagged (**A**) poly-GA, (**B**) poly-AP, (**C**) poly-GR and (**D**) poly-PR constructs at a range of different repeat lengths. Repeat sequences were isolated from DPR constructs by double-digest with BamH1 and EcoR1 followed by gel purification. Band sizes shown as number of bases on left. Repeat-length indicated by numbers above. DNA ladders shown in left hand lanes are Bioline Hyperladders 1 kb+ and 100 bp. E-H Western blots confirming expression of full-length GFP-tagged DPR proteins in HeLa cells, 24 h post-transfection. Predicted sizes of GFP-tagged peptides are: GA1020 157.6 kDa; GR1136 269.2 kDa; AP1024 172.3 kDa and PR1100 278.7 kDa. Antibodies used were Santa-Cruz anti-GFP (E, F), ProteinTech anti-AP (G) and ProteinTech anti-PR (H). All antibodies diluted 1:1000 in 4% BSA.

Our cloning strategy allowed us to generate DPR constructs with various repeat numbers, including ones with similar size to those observed in patients i.e. over one thousand repeats (Table 1). We were able to generate constructs with 1024 repeats for AP, 1136 for GR, 1100 for PR and 1020 for GA. With western blot analysis GA and PR expressed as a single band of the predicted size. GR produced a smear from the predicted size to higher molecular weight species. AP also express at the predicted size with several other additional larger bands presumably resulting from post translational modification (Fig. 1). Unfortunately, the GP construct appeared resistant to expansion of repeat numbers and we were unable to generate a construct over 22 repeats.

**Table 1.** Number of DPR repeats obtained from our cloning strategy

GFP-tagged DPR				
GA	AP	GR	PR	GP
36	22	36	36	22
102	43	63	55	
204	64	71	110	
510	192	107	220	
816	256	142	440	
1020	512	284	550	
	1024	568	880	
		1136	1100	

**Figure 2.**
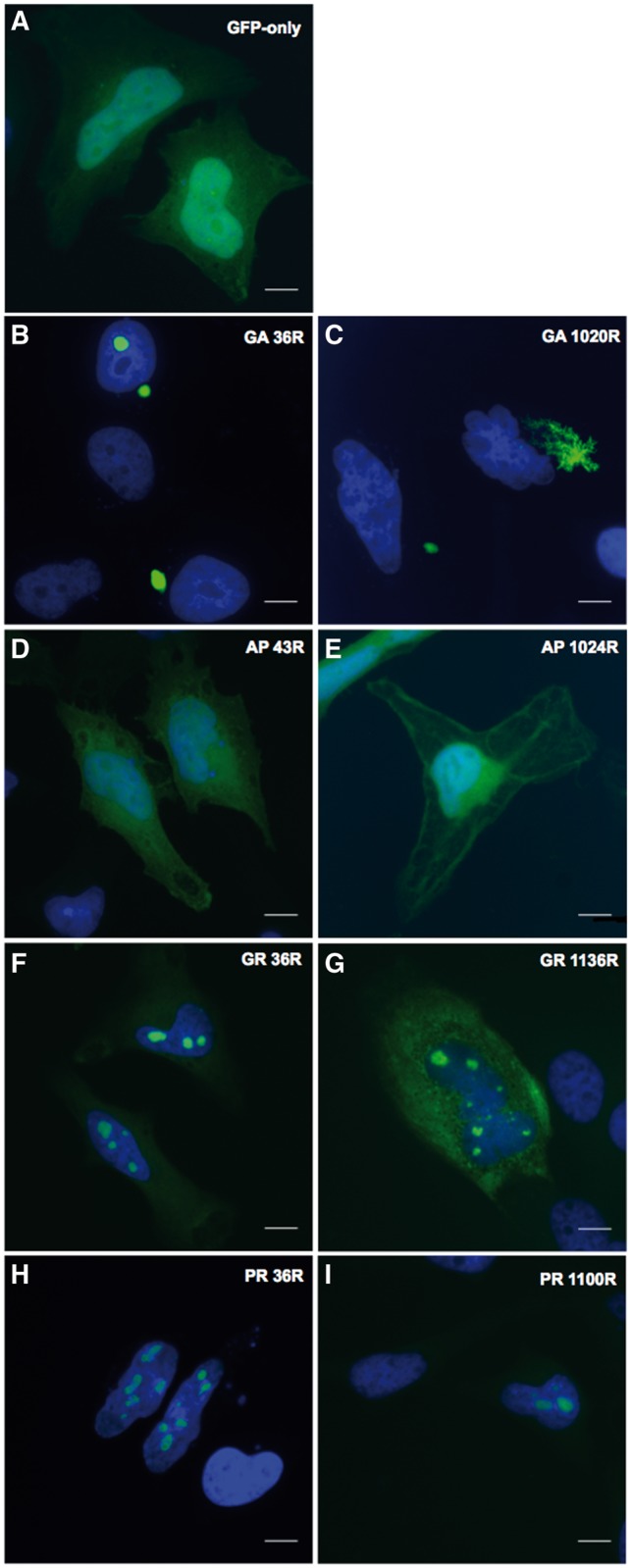
Differential distribution of GFP-tagged DPRs (green) in HeLa cells at 24h post-transfection, or pEGFP-N1 expression as a GFP-only control **(A)**. Representative images are shown at short and long repeat-lengths for Poly-GA **(B,C)**, poly-AP **(D-E)**, poly-GR **(F-G)** and poly-PR **(H-I)**. Repeat lengths are denoted by the letter ‘R’. Nuclei are labelled in blue (DAPI). Images captured at x60 magnification. Scale bars represent 15 µm.

**Figure 3.**
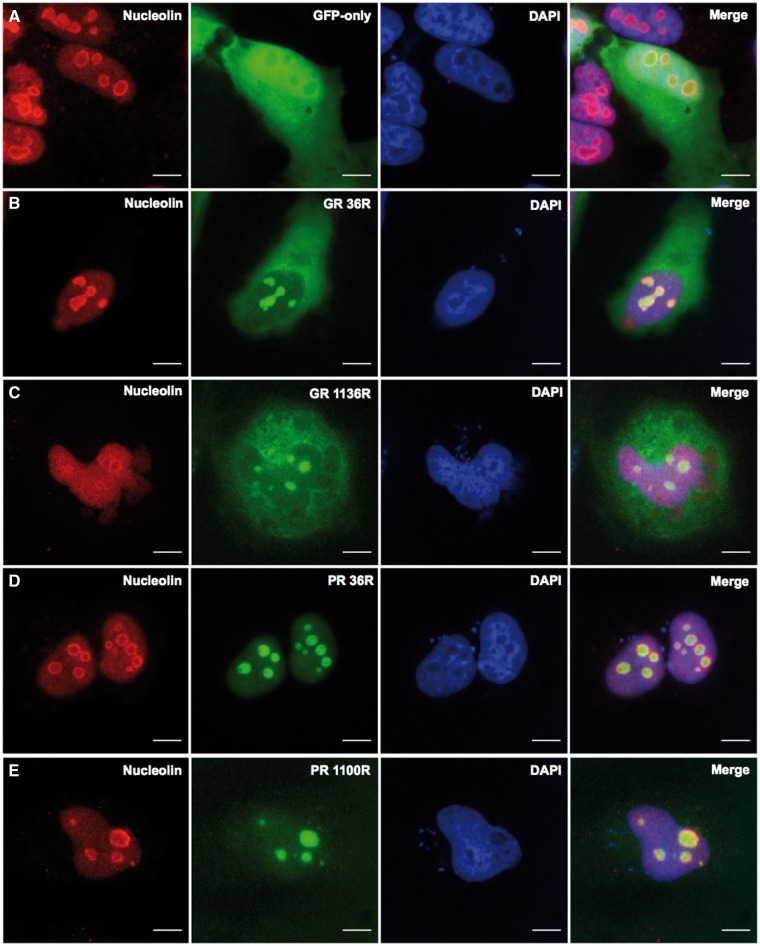
Arginine-rich DPRs translocate to the nucleolus in HeLa cells, as demonstrated by co-localization with the nucleolar marker protein, nucleolin (red). Nucleolin staining in GFP-only control cells expressing pGFP-N1 (green) is shown in **A**. Poly-GR (green) co-localizes with nucleolin at the short and long lengths of 36 and 1136 repeats (**B** and **C,** respectively). Poly-PR (green) also co-localizes with nucleolin at the short and long lengths of 36 and 1100 repeats (**D** and **E**). Images captured at x60 magnification, 24 h post-transfection. Scale bars represent 15 µm.

Transfection of short GA repeats (36) into Hela cells produced discrete, spherical inclusions (Fig. 2B). Whereas, transfection of 1020 GA repeats produced a markedly different phenotype with much more of an irregular fern-like or stellate shaped inclusion being present. Some cells, however, did not form inclusions but displayed a diffuse cytoplasmic GFP presentation. These occurred at all DPR lengths. The shorter AP construct (43 repeats) produced a phenotype similar to GFP alone but the longer AP peptide (1024 repeats) appeared to co-localize with the cellular membrane and cytoskeleton. Both shorter arginine-rich DPRs (GR and PR 36 repeats) localized to nucleolar-like structures. This phenotype remained unchanged with the longest PR (1100 repeats), however, with the longer GR DPR (1136 repeats), diffuse cytoplasmic staining also became a common observation. Removal of the GFP tag had no effect on inclusion formation (Supplementary Material, Fig. S2). To confirm the arginine-rich DPRs were indeed co-localizing to the nucleoli we co-stained cells with the nucleolar marker, nucleolin. Figure 3 demonstrates staining of nucleolin around both the short and long arginine rich DPRs, confirming these are indeed localized to the nucleoli.

**Figure 4.**
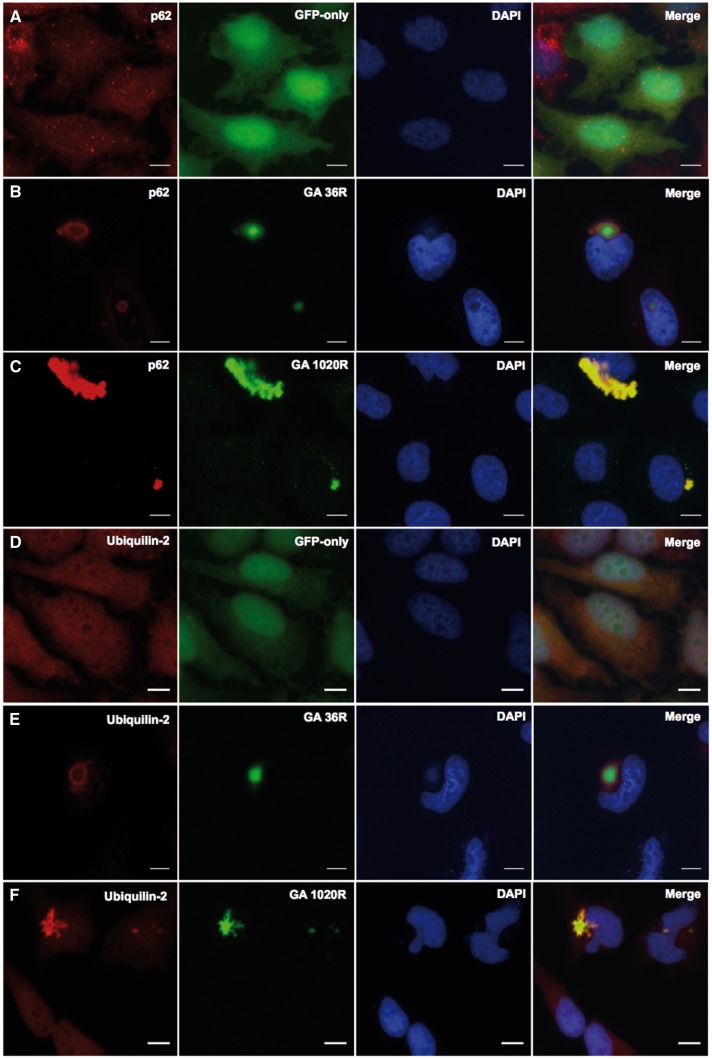
Co-localization of poly-GA with components of the ubiquitin-protease system in HeLa cells, 48h post-transfection. GFP-tagged GA_36_ and GA_1020_ (green) co-localised with **(B, C)** p62 (red) and **(E, F)** ubiquilin-2 (red). The distribution of p62 and ubiquilin-2 in GFP-only control cells transfected with pEGFP-N1 are shown in panels **A** and **D** respectively. Nuclei are labelled in blue (DAPI). Images captured at x60 magnification. Scale bars represent 15µm.

It is known that DPR inclusions in C9orf72 related disease can also contain ubiquilin-2 and p62. Therefore, to investigate whether our DPR expression constructs also recapitulate these features of human disease, we labelled cells transfected with GA (1020 repeats) with antibodies to these proteins. As can be seen in Fig. 4, the GA DPR clearly co-localizes with both p62 and ubiquilin-2 and therefore does recapitulate these features of human pathology. The arginine-rich and AP DPRs did not co-localize with these proteins (data not shown).

### Investigation of nucleolar stress

**Figure 5.**
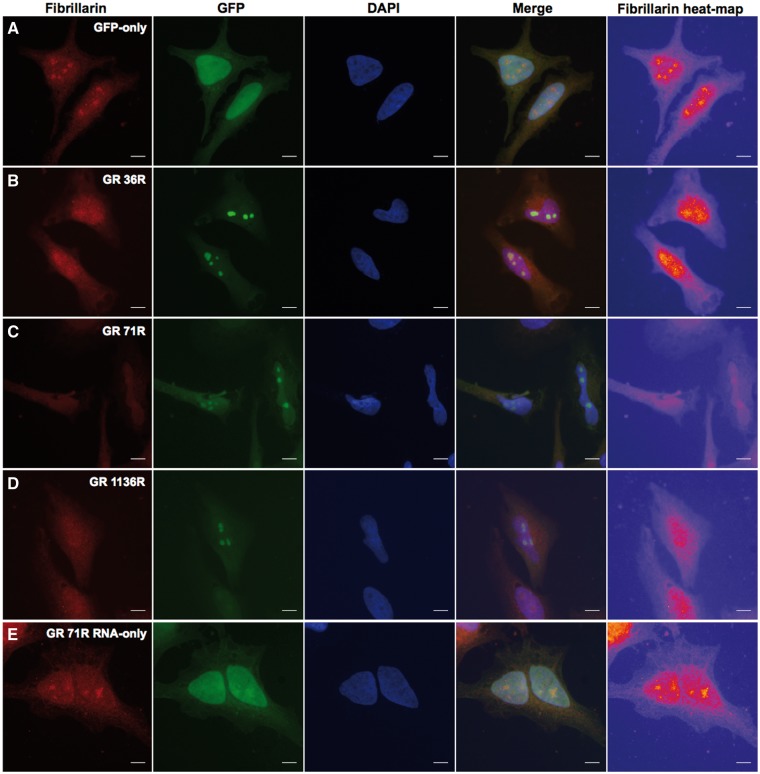
Poly-GR peptide, not RNA, causes a nucleolar stress phenotype. Heat-maps showing loss or re-distribution of fibrillarin in cells expressing nucleolar GR_1136_ compared to GFP-only control. Yellow ‘hot-spots’ indicate areas of high intensity fibrillarin staining, with lower signal areas displayed in red, purple and blue, in decreasing order. **(A)** Fibrillarin forms punctate nuclear ‘hot-spots’ corresponding to the nucleoli in cells expressing pEGFP-N1. Yellow hot-spots become more diffuse with increasing repeat-length of poly-GR from GR_36_**(B)** until no yellow is visible at GR_1136_**(D)**. **(E)** Expression of an RNA-only poly-GR construct did not affect fibrillarin distribution. Images captured in HeLa cells at x60, 48 h post-transfection. Scales bars represent 15 µm.

It has been claimed that arginine-rich DPRs can lead to nucleolar stress. Therefore, to establish if this was the case in our model system we stained Hela cells transfected with GR DPR of increasing lengths with an antibody against the nucleolar protein, fibrillarin, the loss of which is indicative of nucleolar stress. Images were processed to produce a heat map for fibrillarin. With expression of 36 repeats (Fig. 5B), the nucleoli appeared slightly diffuse, but with longer repeats, 71 and 1136 (Fig. 5C + D), fibrillarin staining was completely lost, confirming nucleolar stress. In addition, we demonstrate this is due to DPR protein alone, and not RNA. Figure 4E is a GR construct where the start methionine has been removed using site directed mutagenesis. Therefore, this will lead to DPR mRNA being produced but not protein. However, there is a start methionine for the GFP so this protein is produced. Figure 4E shows that in the presence of DPR mRNA, but not protein, fibrillarin expression is unaffected indicating that nucleolar stress is not occurring.

The data in Fig. 5 suggested that there might be a length dependent effect on DPR expression. Given that many of the reported C9orf72 models use repeat lengths smaller than those observed in patients we investigated this further. The effect of length on DPR phenotype was investigated using electrophysiology.

### Electrophysiology

**Figure 6.**
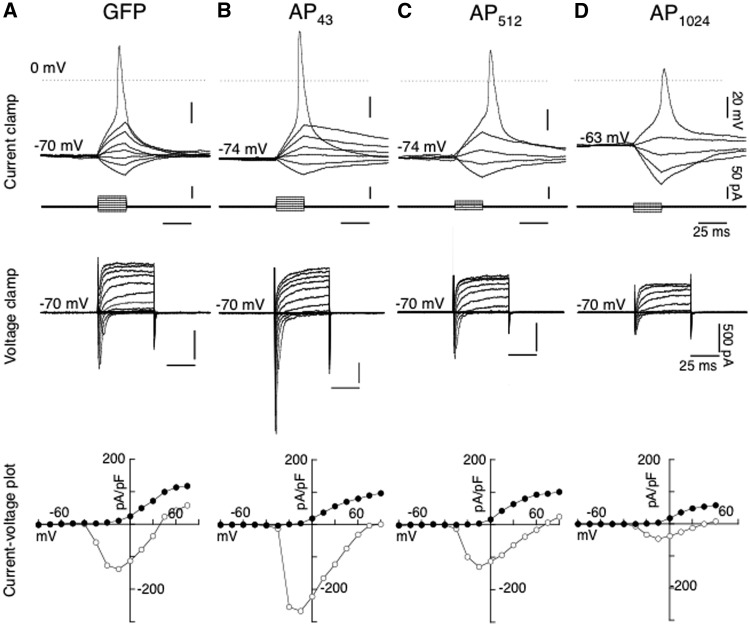
Electrophysiological profiles from differentiated SH-SY5Y cells transfected with GFP alone and AP_n_-GFP, under whole cell current and voltage clamp recording mode. *Top traces* depicted whole cell current clamp recordings in representative cells transfected with GFP *(****A****)*, AP_43_-GFP *(****B****)*, AP_512_-GFP *(****C****)* and PA_1024_-GFP *(****D****)*, where voltage traces in response to hyperpolarizing and depolarizing steps (25 ms duration, 10 pA increment, corresponding traces *shown below*). Depolarizing steps to a threshold value elicited an action potential from their respective holding potentials. *Middle traces* show the current responses under whole cell voltage clamp mode of the corresponding cells shown in GFP *(****A****)*, AP_43_-GFP *(****B****)*, AP_512_-GFP *(****C****)* and PA_1024_-GFP *(****D****)* at a command holding potential of -70 mV to a series of hyperpolarizing and depolarizing pulses (50 ms, 10 mV increment), and (*bottom traces*), the resultant I-V plots of peak current (pA/pF) (open circle) and currents (pA/pF) at the end of 50 ms (closed circle).

GFP plasmid: GFP plasmids were transfected into differentiated SH-SY5Y cells, as a control. As expected, transfection with only GFP plasmid resulted in a diffuse cytosolic distribution. Under whole-cell current clamp mode, membrane time course was estimated with hyperpolarizing pulses (10 pA, 25 ms duration) from resting potential, yielding an average value of 26.3 ± 3.9 ms (*n =* 11) (Supplementary Material, Table S1). Whereas, action potentials were generated with a series of increasing depolarizing voltage steps (25 ms, 10 pA increment) from holding around −70 mV (Fig. 6A), when threshold was reached. Pooled data from 11 cells generated an average spike threshold value of − 44.0 ± 1.5 mV, spike width of 7.2 ± 0.8 ms, spike amplitude of 60.6 ± 3.3 mV and spike overshoot (above 0 mV) by of 16.6 ± 3.5 mV (Supplementary Material, Table S1). Under whole-cell voltage clamp mode, GFP control cells exhibited an average holding current at − 60 mV of − 19.6 ± 3.8 pA (*n =* 11). A series of hyperpolarizing and depolarizing current steps (50 ms) from −80 to +50 mV, from a command holding potential of − 70 mV, evoked a series of fast inward current and slowly developing outward current (Fig. 6A). The inward current underlying activation of sodium channels can be clearly revealed by plotting the peak and sustained current density (pA/pF) as a function of voltage (Fig. 6A). The inward current was activated from a potential of approximately −40 mV and peaked at − 10 mV, with a corresponding increase in the outward conductance. Pooled data of hyperpolarizing pulses to − 80 mV from a holding potential of − 70 mV produced average input resistance values of 2.4 ± 0.3 GΩ, whereas a depolarizing step to +30 mV, underlying the delayed potassium outward rectifier yielded an average value of − 73.2 ± 8.6 pA/pF (*n =* 11) (Supplementary Material, Table S1). The passive data of time constant, input resistance and cell capacitance in RA-differentiated control SH-SY5Y cells in the present study were comparable with those reported by Johansson (1994).

The inward sodium current can be completely blocked by tetrodotoxin (TTX, 0.5 μM, data not shown) whereas the outward current responsible for the repolarizing phase of the action potential, consisting of a delayed rectifier can be partially blocked by low concentration of tetraethylammonim chloride (TEA, 100 μM, data not shown).

Alanine-Proline (AP): Differentiated SH-SY5Y cells transfected with APn-GFP. The expression of AP peptides at all lengths tested (43 to 1024) was similar to GFP (Fig. 6A), having a diffuse cytosolic distribution. Examples of current and voltage clamp recordings from cells expressing repeats of 43, 512 and 1024, are shown in Figs 6B, C and D, respectively. Cells expressing repeats of 43 exhibited large action potentials (Fig. 6B), and there was a gradual decrease in spike amplitude with increasing repeats to 512 (Fig.6C) and 1024 (Fig. 6D), with a small decrease in the amplitude of outward current amplitude to +30 mV. This gradual inhibitory effect of AP with expanding repeats is exemplified by current-voltage density plots of the illustrated cells in Fig. 6B and C. Pooled data from cells expressing all repeat-lengths of AP are represented in Supplementary Material, Table S1, along with those of GFP control. Cells expressing AP at 43 repeats exhibit larger spike amplitude and overshoot with values of 71.4 ± 8.7 mV and 24.5 ± 8.5 mV (*n =* 5) compared to those obtained with GFP of 60.6 ± 3.3 mV and 16.6 ± 3.5 mV (*n =* 11), respectively. However, following statistical analysis, these and other parameters recorded were considered not to be statistically different to GFP control (see Supplementary Material, Table S1). It was also apparent that with the larger AP construct of 1024 repeats, there was a noticeable reduction in viability of patched cells from the increase of green, shrunken dead cells in the cultures. Insoluble inclusions were never observed with any of the AP repeat constructs.

**Figure 7.**
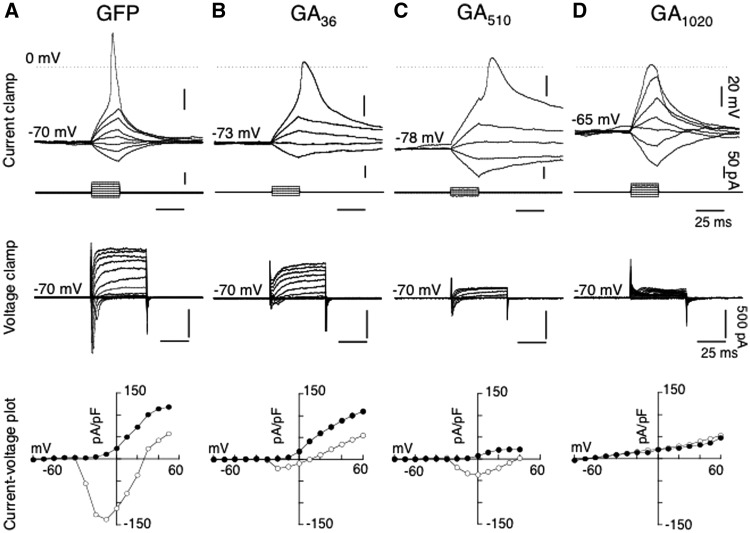
Comparison of electrophysiological profiles from differentiated SH-SY5Y cells transfected with GFP alone and GA_n_-GFP, under whole cell current and voltage clamp recording mode. *Top traces* illustrated whole cell current clamp recordings in a control cell transfected with GFP *(***A***, shown in**Figure 1*), and representative cells with cytoplasmic inclusions following transfection with GA_36_-GFP (**B**), GA_510_-GFP (**C**) and GA_1020_-GFP (**D**), where voltage traces in response to hyperpolarizing and depolarizing steps (25 ms duration, 10 pA increment, corresponding traces *shown below*), revealing the effect of the increasing repeat lengths of GA_n_-GFP on action potential waveform from their respective holding potentials. *Middle traces* show the current responses under whole cell voltage clamp mode of the corresponding cells shown in GFP (**A**), AP_43_-GFP (**B**), AP_512_-GFP (**C**) and PA_1024_-GFP (**D**) at a command holding potential of − 70 mV to a series of hyperpolarizing and depolarizing pulses (50 ms, 10 mV increment), and (*bottom traces*), the resultant I–V plots of peak current (pA/pF) (open circle) and currents (pA/pF) at the end of 50 ms (closed circle).

Glycine-Alanine (GA): Differentiated SH-SY5Y cells transfected with GAn-GFP displayed stellate-like cytoplasmic inclusions. At 36 repeats, cells with inclusions were rarely observed in the cultures and only two cells could be recorded (2/12): one cell lacked action potentials and the other exhibited attenuated action potentials in terms of reduced amplitude, overshoot and with increase spike width (Fig. 7B) compared to GFP control (Fig. 7A) (Supplementary Material, Table S2). Increasing repeat number to 510 yielded more cells with inclusions (6/10) and of the 6 cells, 3 cells exhibited attenuated action potentials (Fig. 7C), together with an increase in spike duration as a result of the decrease in outward conductance (as depicted in the current-voltage density plots; Fig. 7). With 1020 repeats, no action potential could be evoked in 3/5 cells (Fig. 7D). Pooled data are shown in Supplementary Material, Table S2, showing that cells expressing GA510-GFP had significant differences (*P <* 0.05) in their electrophysiological profile compared to GFP control. Repeats of 1020 produced more cells without action potential to compare to GFP control (Supplementary Material, Table S2). With repeats of 1020 and to some extent repeats of 510, it was apparent that less viable cells with inclusions were present in cultures, and this was reflected in the low numbers of cells patched, due to an increase in dead cells containing inclusions.

**Figure 8.**
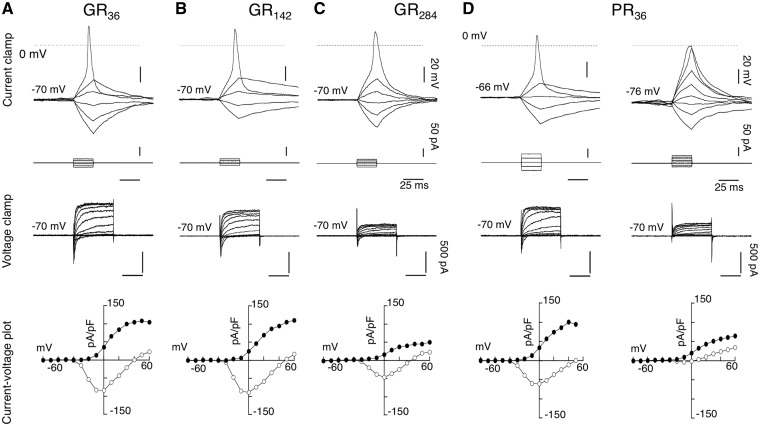
Electrophysiological profile of arginine-containing DPRs: Glycine-arginine (GR) and proline-arginine (PR) proteins in differentiated SH-SY5Y cells. (**A**), a representative cell expressing GR_36_-GFP, (**B**), a cell expressing GR_142_-GFP, (**C**) a cell expressing GR_284_-GFP and (**D**) representative cells expressing PR_36_-GFP. (*Top traces*), current clamp responses (25 ms, 10 pA increment) elicited action potential waveform in response to a threshold stimulation. (*Middle traces*) showing the corresponding current traces under voltage clamp from −70 mV (50 ms, 10 mV increment), and (*bottom traces*), the resultant I-V plots of peak current (pA/pF) (open circle) and currents (pA/pF) at the end of 50 ms (closed circle). *Note*, that recordings with PR even at the lowest repeats of 36 repeats evoked two types electrophysiological profiles, either ‘normal’ or ‘lack’ of action potential.

Glycine-Arginine (GR): GRn-GFP constructs exhibited a characteristic diffuse cytoplasmic GFP distribution of the cell soma, together with GFP positive nucleoli at all repeat lengths tested in differentiated SH-SY5Y cells. Similar cytoplasmic localization of GR80 has recently been reported (23), although these authors did not note any nuclear localization. There were less viable cells for patching when GR repeat length was increased from 36 to 142, and this was reduced further with increasing length to 284 repeats. No viable cells could be patched at repeat lengths greater than 284. As illustrated in Fig. 8 in representative cells of GR36-GFP (Fig. 8A), GR124-GFP (Fig. 8B) and GR284-GFP (Fig. 8C), the electrophysiological profile and parameters of viable cells expressing GR36-GFP to GR284-GFP were similar to those of GFP-control (Supplementary Material, Table S3). However, the GR constructs exhibited a length-dependent effect on cell excitability, with expression of 284 repeats significantly (*P <* 0.005) reducing the outward conductance evoked with depolarizing pulses to +30 mV, with a value of 26.0 ± 8.3 pA/pF (*n =* 4) compared to that of control GFP (73.2 ± 8.6 pA/pF; *n =* 11).

Proline-Arginine (PR): In contrast to GR, PR constructs were expressed entirely in the nucleus, with diffuse (soluble) nucleoplasm staining along with expression in the nucleoli. The intensity of the GFP signal in nucleoli with PR constructs was weak, and, unlike GR, it could be readily photobleached with continued exposure to ultraviolet light. Although there were no apparent differences in the DPR localization in cells expressing PR constructs, they did, however, exhibit two types of electrophysiological profiles following depolarizing pulses: (1) where an action potential could be evoked and (2) where no action potential was evoked, as shown with PR36-GFP (Fig. 8D). This was reflected in the respective current-voltage density plots (Fig. 8D), clearly showing the lack of inward current and reduced outward current, although the reduction in outward current was not seen in most cells tested.

The pooled data revealed that with PR constructs, the cells were more depolarized as they exhibited significantly (*P <* 0.05) higher holding current at -60 mV and had reduced overshoot, without statistical significant effects on threshold of the action potential or on the outward conductance to +30 mV (Supplementary Material, Table S4). Similar profiles were recorded from all repeat-lengths of PR (36 to 550) (Supplementary Material, Table S4). No cells expressing PR at 1100 repeats were viable for patch-clamping.

## Discussion

Since the original identification of the hexanucleotide repeat expansion in C9orf72 as the cause of FTLD and ALS linked to chromosome 9 in 2011, there have been numerous reports of attempts to model this in various cell types and animal species. While the exact numbers of repeats needed to cause clinical disease in humans are not yet established, the vast majority of confirmed cases have repeat sizes that range from 500 to in excess of 4000 (3,24,25). Given the immense technical challenges of manipulating large numbers of DNA repeats with a 100% GC content, the majority of models have used small numbers of repeats, many of which develop a neurodegenerative phenotype. The transgenic mouse reported by Chew *et al*. with 66 repeats had behavioural abnormalities with DPR and TDP-43 pathologies. However, the mice reported by O'Rourke *et al.* (26) and Peters *et al*. (27) with large numbers of repeats failed to recapitulate the clinical and pathological features of FTLD/ALS. Not all DPR species were reported being present in these mice and this may explain the lack of phenotype. Nevertheless, as it is known that 30 hexanucleotide repeats in C9orf72 can produce DPR inclusions without TDP-43 pathology, neurodegeneration or clinical symptoms in humans (21), models expressing repeat numbers that do not at least approach the size seen in human disease are of questionable physiological relevance. Therefore, to address this issue for the first time, we have generated expression constructs for DPRs with repeat numbers that do match the size seen in patients. When expressed in cells, these recapitulate many of the features exhibited in human C9orf72 pathology.

In the present study, we have selected to use differentiated SH-SY5Y cells as our model of neurons as they exhibit voltage-gated sodium and potassium channels, which are responsible for action potentials and therefore crucial for neuronal activity and cell survival. Furthermore, differentiated SH-SY5Y cells may be easily transfected with DPR plasmids using Lipofectamine, which make them a useful model to evaluate the functional impact of DPRs on cellular excitability using whole-cell recordings.

We transfected differentiated SH-SY5Y cells with DPR plasmids of varying repeat-length or GFP-only control, and the parameters of their passive and active properties were evaluated. AP constructs up to 1024 repeats in length were observed to have a small, length-dependent effect on cellular excitability. similar to previous report by Mizielinska *et al*. (2014) (21) where the lack of neurotoxicity was found with constructs up to 100 repeats, nevertheless a length dependent effect on voltage dependent conductances was still observed (17). Wen *et al*. (2014) reported marginal neurotoxicity with AP220-GFP (16). Taken together, these results indicate that AP DPR expression has little effect on cellular excitability until the repeat-length reached over 1000. It is therefore possible that models expressing AP at short repeat-lengths may not capture subtle, length-dependent phenotypes which may contribute to neuronal dysfunction in FTLD/MND.

Expression of GA proteins produced cytoplasmic stellate-shaped inclusions which increased in size and formed a characteristic fern-like structure at longer repeat-lengths. Expression of GA at various repeat-lengths reduced sodium conductance in differentiated SH-SY5Y cells, whilst reductions in potassium conductance were observed only at longer repeat-lengths. Neurotoxicity of GA has been previously reported in cells expressing short repeat-lengths of 50 and 175 units, with endoplasmic reticulum stress and caspase-3 activation observed in cells containing GA inclusions (29). However, our data suggest that whilst GA repeats may confer cytotoxicity at short repeat-lengths, a more severe phenotype is observed once repeats reach physiological size.

Although there are still conflicting reports on the contribution of GA proteins to cellular neurodegeneration, there is widespread agreement that the arginine- containing DPRs are mediators of neurotoxicity (16,17,28,29). In our cellular models, both GR and PR translocated to the nucleolus, where GR was found to cause nucleolar stress in a length-dependent manner. Constructs with a higher number of repeats were more toxic, as demonstrated by an increased presence of dead GFP-labelled cells in our culture and a decrease in our ability to record from transfected cells. The present study indicates that GR exerted length-dependent neurotoxicity, with expression of 284 repeats causing a significant reduction in the outward potassium current (5/5 cells), along with cells in which no sodium action potential could be evoked (3/5 cells). No data are included for repeat-lengths above 284 since no viable cells were available for patch-clamping above this length, indicating a more severe toxic phenotype. Similar length-dependent neurotoxicity with GR proteins has been reported by Wen *et al*. (2014) (16) and Mizielinska *et al*. (2014) (17). In contrast, reduction in cellular excitability was observed in cells expressing PR at all repeat-lengths from 36 to 550 repeats (Supplementary Material, Table S4), suggesting that PR toxicity is severe regardless of length. However, no cells expressing PR at 1100 repeats were viable for patch-clamp recording, indicating that PR toxicity may be even further exacerbated at physiological lengths of over 1000 repeats. PR expression was observed to preferentially inhibit sodium channels (Fig. 8D). In summary, we have produced a series of cellular models that recapitulate DPR pathology observed in humans. Furthermore, our findings provide for the first time, clear evidence that DPRs have a length-dependent ability to modulate both sodium and potassium conductances, which are important for the initiation and propagation of action potentials. The dysfunction of these conductances is most likely a contributing factor to neuronal death. Given that both shorter and longer DPRs both produce intracellular inclusions but the effects on electrophysiology were only seen with DPRs of a length seen in human disease, our findings suggest that DPR models should be of this larger size to ensure physiological relevance.

## Materials and Methods

### Generation of constructs for dipeptide expression

DNA sequences coding for each DPR were designed using alternative codons in a semi randomized fashion to encode the same amino acids found in the DPR but with a reduction of repetitiveness G4C2 RNA (sequences shown in Supplementary Material, Table S1). An ATG start codon was placed at the beginning of each DPR sequence. Restriction sites for the type-2S restriction endonuclease enzymes, FokI and BbsI, were placed at the beginning and end of the DPR sequence, respectively, to cleave at either end without additional cleavage within the repeat section. Short DPR constructs were initially manufactured by Eurofins MWG Operon at lengths of 36 repeats for poly-GA, -GR and –PR and 22 repeats for poly-AP and –GP, where one repeat unit is considered to be six bases/two amino acids. The sequence, including DPR sequence, start codon and type-2S restriction sites was cloned into the pEGFP-N1 vector (ClonTech) using BamHI and EcoR1 restriction sites, creating constructs for expression of each DPR with a C-terminal GFP-tag, driven by the CMV promoter. The DPR sequences were then elongated in a step-wise manner; DPR sequences were isolated by digesting with FokI and BbsI, and ligated into BbsI-linearized DPR vector to double repeat-length (Supplementary Material, Fig. S1). This process was repeated until physiologically-relevant repeat-lengths of over 1000 units were obtained (Fig. 1). The empty pEGFP-N1 vector was used as a GFP-only control throughout, since all DPRs were expressed using this vector.

To confirm the GFP-tag did not affect the subcellular localization of DPRs, ‘untagged’ versions of the DPR vectors were generated using site-directed mutagenesis to introduce a stop codon between the repeat sequence and GFP gene. Site-directed mutagenesis was performed using the QuikChange Lightning kit (Agilent Technologies) and the following primers:

Poly-GA forward: CCG GTG CTG TCT TCC AAC GGG ATC CAC CG

 Poly-GA reverse: CGG TGG ATC CCG TTG CAA GAC AGC ACC GG

Poly-AP forward: GGC CCC TGT CTT CCA ACG GGA TCC ACC

 Poly-AP reverse: GGT CGA TCC CGT TGG AAG ACA GGG GCC

Poly-PR forward: CCC GCG AGT CTT CCA ACG GCA TCC ACC

 Poly-PR reverse: GGT GGA TCC CGT TGG AAG ACT CGC GGG

Poly-GR forward: GCG GCA GAG TCT TCC AAC GGG ATC CAC CG

 Poly-GR reverse: CGG TGG ATC CCG TTG GAA GAC TCT GCC GC

Poly-GP forward: GGG GCC TGT CTT CCA ACG GGA TCC ACC

 Poly-GP reverse: GGT GGA TCC CGT TAG AAG ACA GGC CCC

To generate an ‘RNA-only’ version of the GFP-tagged GR71 construct, the start codon preceding the DPR sequence was removed using the QuikChange Lightning kit (Agilent Technologies). Whilst removal of the start codon would be sufficient to prevent translation of this sequence, there is also a stop codon several bases upstream of the amended bases. A start codon in the GFP sequence was retained, allowing expression of GFP protein as a marker of transfected cells.

Forward: CCCGACCGCTCCTCTGCAA TTGGACTACAT CCG AATTCGAAG

Reverse: CTTCGAAT TCGGATGTAGT CCAATTGC AGA GGA C GCGGTCGGG

### Validation of DPR constructs

Sequencing was performed on both the forward and reverse strands by the University of Manchester Sequencing Service using the commercially available CMV forward and pEGFP-N1 reverse primers flanking the multiple cloning site in the pEGFP-N1 vector. Given the length and repetitive nature of the DPR plasmids, sequencing of the entire DPR sequence was not technically possible for the majority of constructs above ∼100 repeats. All constructs were therefore validated in 3 ways: (i) the ends of the repeat region were sequenced using the CMV forward and pEGFP-N1 reverse primers, which was successful for ∼100 repeats in both directions, (ii) The repeat sequences were isolated from the plasmid by restriction digest with BamHI and EcoRI (Supplementary Material, Fig. S1) and agarose gel electrophoresis was performed on digested plasmids to determine repeat-length and (iii) all plasmids were expressed *in vitro* to ensure C-terminal GFP expression was observed. This ensured that that no frame-shift mutations had been introduced within the repeat, as this would prevent translation of a C-terminal tag.

### Cell culture and transfection

HeLa cells (ECACC #93021013) were maintained at 37 °C with 5% CO2 in Dulbecco’s Modified Medium (DMEM; Sigma). DMEM was supplemented with 10% v/v foetal calf serum (Gibco), 2 mM L-glutamine (Sigma), 100 U/ml penicillin and 100 µg/ml streptomycin (Sigma) prior to use. HeLa cells were seeded on HCl-treated glass 22 mm coverslips in 6-well plates in DMEM at appropriate densities to achieve ∼70% confluency following overnight incubation at 37 °C. Transient transfections were performed using FuGene HD (Promega; 7.2 µl per well) and 800 ng/well DPR construct or empty pEGFP-N1 vector as a GFP-only control. The media was replaced ∼4 h after transfection to prevent excessive toxicity.

SH-SY5Y cells were maintained in non-confluent monolayer in MEM growth medium (MEM-GM, Life Technologies, UK), consisting of Earle’s Minimal Essential Medium (MEM), supplemented with 10% foetal calf serum, penicillin (100 U/ml) and streptomycin (100 µg/ml), L-glutamine (2 mM) and 1% Non-essential amino acids in a 95% air/5% CO_2_ humidified incubator at 37°C. Cells were subcultivated twice a week, when 70% confluency was reached. 24 h before differentiation, SH-SY5Y cells were plated out on poly-L-lysine coated coverslips in EMEM-GM for cell adhesion, and differentiation induced with 3 µM all-trans-retinoic acid (Sigma) in N2-growth medium (N2-GM) consisting of DMEM/F12, 2 mM L-glutamate, penicillin (100 U/ml) and streptomycin (100 µg/ml) and 1% N2-supplement. N2-GM + RA medium was replaced every 3 days, and continued for 14–21 days.

For electrophysiology, differentiated SH-SY5Y cells were transiently transfected for 4 h with 250 ng/ml of dipeptide repeat constructs using Lipofectamine 2000 (Life Technologies) in accordance with manufacturer’s instructions. For control, the pEGFP-N1 vector was transfected at equal concentrations of 250 ng/ml. Recordings were made 48–78 h after transfection.

### Immunofluorescence

HeLa cells were prepared for immunofluorescence imaging at 24 or 48 h post-transfection. Cells were washed with warm phosphate buffered saline (PBS), fixed in 4% paraformaldehyde w/v in PBS at room temperature for 20 min, permeabilized with 0.1% TritonX-100 v/v in PBS for 10 min at room temperature and blocked in 1% fish skin gelatin v/v in PBS for 1h. All antibody incubations were performed at room temperature for 30 min. Coverslips were washed 2-3 times with PBS between each step. The following primary antibodies were used: α-p62 (Abcam; 1:50), α-ubiquitin (Abcam; 1:400), α-ubiquilin-2 (Abnova; 1:50), α-nucleolin (ProteinTech; 1:200), α-fibrillarin (Abcam; 1:1000), α-poly-GA (ProteinTech; 1:100), α-poly-GR (ProteinTech; 1:100), α-poly-AP (ProteinTech; 1:100), α-poly-GP (ProteinTech; 1:100), α-poly-PR (Genscript; 1:100). The following secondary antibodies were used: goat anti-rabbit AlexaFluor-488, donkey anti-rabbit AlexaFluor-594 and goat anti-mouse AlexaFluor-594 (All Life Technologies; 1 drop in 500 µl PBS). NucBlue DAPI nuclear stain for fixed cells (Life Technologies; 1 drop in 500 µl PBS) was either combined with the secondary antibody or incubated separately for 10 min at room temperature. Coverslips were mounted on glass slides using ProLong Gold mounting reagent (Life Technologies). All immunofluorescence was performed in triplicate. Images were captured using an Olympus BX51 upright microscope with a x60 Plan Fln objective and a Coolsnap ES camera (Photometrics). Specific band pass filter sets for DAPI, FITC and Texas Red were used to prevent bleed through between channels. The image capture software used was MetaVue (Molecular Devices), and images were processed and analysed using ImageJ (http://rsb.info.nih.gov/ij; date last accessed October 3, 2016).

### Fibrillarin imaging

Cells expressing nucleolar poly-GR at a range of repeat-lengths, ‘RNA-only’ GR71 or GFP-only control (pEGFP-N1) were selected for imaging without viewing the Texas Red fibrillarin channel, to avoid bias. Images were captured as described above, maintaining a constant exposure time of 500ms for fibrillarin images. Fibrillarin images were converted into heat-maps depicting the relative signal intensities throughout the cell as colour, using ImageJ (http://rsb.info.nih.gov/ij). Areas of the cell which appeared yellow indicated high intensity of fibrillarin staining at that point, ranging through to red, purple and blue as the signal decreased.

### Whole-cell recordings of transfected SH-SY5Y cells

Coverslips were transferred to the recording chamber fixed on the stage of a Zeiss upright Axioskop FS (Zeiss, UK) microscope and continuously superfused at a rate of 4 ml/min with recording solution of the following composition (in mM): NaCl 134, KCl 5, CaCl2 2, MgCl2 1, HEPES 10, D-glucose 10 and the pH adjusted to 7.4 with 5N NaOH and osmolarity of 280-290 mOsml.

Cells transfected with DPRs or GFP-only control were viewed with a x40 water immersion objective (Zeiss Achroplan 0.75W, Ph2) with both fluorescent and Normaski differential interference contrast optics, and digital images of recorded cells were acquired and processed using a Cooled CCD camera (ORCA C10600, Hamamatsu Corporation) running Simple PCI Instruments Analysis software (Hamamatsu).

Electrophysiological recordings were carried out using the tight-seal whole-cell technique (Hamill *et al*., 1981) with a HEKA EPC10 amplifier running Pulse and Pulsefit software (version 8.54: HEKA, USA). Patch electrodes were pulled from borosilicate glass electrodes (WPI, UK) on a vertical puller (PP-830: Narishige Ltd) and had a resistance of 3-5 M&Omega;, when filled with internal solution containing mM): 140 KCl, 4 NaCl, 5 EGTA, 10 HEPES, 4 MgATP, 0.1 NaGTP and pH adjusted to 7.4 with KOH and osmolarity of 280-290 mOsml. Signals were filtered (0.3–10 kHz, Bessel filter of HEKA EPC 9). Subsequent analysis of the acquired data was performed using Pulsefit software (HEKA, USA), and plots generated with Axograph X (John Clements) and KaleidaGraph (Synergy software, USA).

All recordings were performed at room temperature (20–23°C). Differentiated SH-SY5Y cells are known to exhibit action potentials (Brown *et al*. 1994; Johansson 1994, Toselli *et al*. 1996). In order to evaluate the effect of DPRs on cellular properties, whole-cell voltage recordings were performed under current-clamp conditions. The membrane time constants (τ) of transfected cells were determined from small (0.01-0.02 nA, 50 msec duration) hyperpolarizing steps from a holding potential of −70 to −86 mV. Passing brief (25 msec) depolarizing current pulses (0.05−0.1 nA) through the recording electrode generated single action potentials. Parameters such as spike amplitude, spike threshold, spike overshoot, and duration were measured from the first evoked action potential, and given as mean ± SEM. Spike duration was measured as half of the amplitude from threshold to peak (see Supplementary Material, Table S1). It is worth noting that the terminology used in this study relating to ‘attenuated spike’ refers to a waveform elicited with depolarizing pulses, consistent to an action potential, which did not have an overshoot. However in some cells, depolarizing pulses evoked a family of traces where an action potential was absent (see illustrated example in Fig. 5D), and these were termed as ‘no action potential’, as it was not possible to determine the spike threshold, to evaluate amplitude and duration.

Under voltage-clamp conditions, current-voltage relationships were constructed by series of hyperpolarizing and depolarizing voltage steps (from −80 to +60 mV, with 10 mV, 50 ms increment duration) from a holding potential of −70 mV. Due to high resistance of the cell in current-clamp mode, it was not always possible to determine resting membrane potential accurately, therefore the current required to hold cells at a holding potential of −60 mV was measured, and input resistances were determined from small (−10 mV, 50 ms) hyperpolarizing pulses from −70 mV. In addition, the effect of DPRs on delayed potassium rectifiers was determined with a depolarizing voltage pulse to +30 mV, and expressed as pA/pF (see Table 1). Data were grouped according to the DPR expression pattern, and data pooled were represented as mean ± SEM of n from at least 3 transfections. A statistical analysis was undertaken by one-way analysis of variance (ANOVA) with post hoc two-tailed t-tests, with Instat (Graphpad Software Inc.). Statistical significance was accepted at *P <* 0.05.

### Determination of membrane properties

Differentiated SH-SY5Y cells exhibited action potentials (Brown *et al*. 1994; Johansson 1994, Toselli *et al*. 1996), and to evaluate the effect of the DPRs on cellular property, we performed current-clamp recordings where voltage responses to current stimuli were recorded to be able to evaluate the parameters of the action potential and passive properties of the cells. The membrane time constant (τ) of transfected cells was determined from small (0.01−0.02 nA, 50 msec duration) hyperpolarizing steps from holding potential of −70 to −86 mV. Passing brief (25 msec) depolarizing current pulses (0.05−0.1 nA) through the recording electrode generated single action potential following threshold stimulation. Parameters such as spike amplitude, spike threshold, spike overshoot, and duration were measured from the first evoked action potential, and given as mean ± SEM. Spike duration was measured as half of the amplitude from threshold to peak (see Supplementary Material, Table S1). It is worth noting that the terminology used in this study relating to ‘attenuated action potential’ refers to a waveform elicited with depolarizing pulses, consistent to an action potential, which did not have an overshoot. However in some cells, depolarizing pulses evoked a family of traces where an action potential was absent (see illustrated example in Fig. 5D), and these were termed as ‘no action potential’, as it was not possible to determine the spike threshold, to evaluate amplitude and duration.

Under voltage clamp conditions, current-voltage relationships were constructed by series of hyperpolarizing and depolarizing voltage steps (from −80 to +60 mV, with 10 mV, 50 ms increment duration) from holding potential of −70 mV. Due to the high resistance of the cell in current clamp mode, it was not always possible to determine resting membrane potential accurately, therefore we measured the current required to hold cells at a holding potential of −60 mV, and input resistances were determined from small (−10 mV, 50 ms) hyperpolarizing pulses from −70 mV. In addition, effect on the delayed potassium rectifier was determined with a depolarizing voltage pulse to +30 mV, and expressed as pA/pF (see Table 1). Pooled data were represented as mean ± SEM of *n* determinations of each construct from at least 3 transfections, and statistical analysis was undertaken by one-way analysis of variance (ANOVA) with post hoc two-tailed *t*-tests, with Instat (Graphpad Software, Inc., La Jolla, CA, USA). Statistical significance was accepted at *P <* 0.05.

## Supplementary Material

Supplementary Material is available at *HMG* online.

## Supplementary Material

Supplementary Data
