# Supplementary material for: Modelling C9orf72 dipeptide repeat proteins of a physiologically relevant size
Source: Hum Mol Genet. 2016 Oct 23;25(23):5069–82. doi: 10.1093/hmg/ddw327 (PMC5886041; doi:10.1093/hmg/ddw327)
Supplement: Supplementary Data [file ddw327_supp.docx]

## Supplemental info

**Alternative codon sequences:**

**Poly-GA:** ATG GGT GCT GGC GCG GGA GCA GGC GCT GGT GCT GGT GCA GGA GCG GGT GCG GGA GCT GGT GCC GGC GCA

**Poly-GP:** ATG GGG CCT GGC CCC GGT CCC GGT CCT GGT CCA GGC CCT GGA CCT GGC CCA GGA CCC GGC CCA GGA CCA GGT

**Poly-GR:** ATG GGT CGT GGA CGT GGA CGA GGT CGA GGT CGA GGT CGT GGA CGT GGT CGA GGT CGA GGT CGT GGA CGT

**Poly-PR:** ATG CCG CGA CCT CGA CCG CGG CCA CGC CCA CGC CCT CGG CCC AGA CCA CGT CCT AGG CCC AGA CCC AGA CCC

**Poly-AP:** ATG GCT CCT GCA CCA GCA CCT GCT CCT GCA CCA GCA CCA GCC CCT GCT CCT GCT CCA GCA CCA GCT CCT GCA


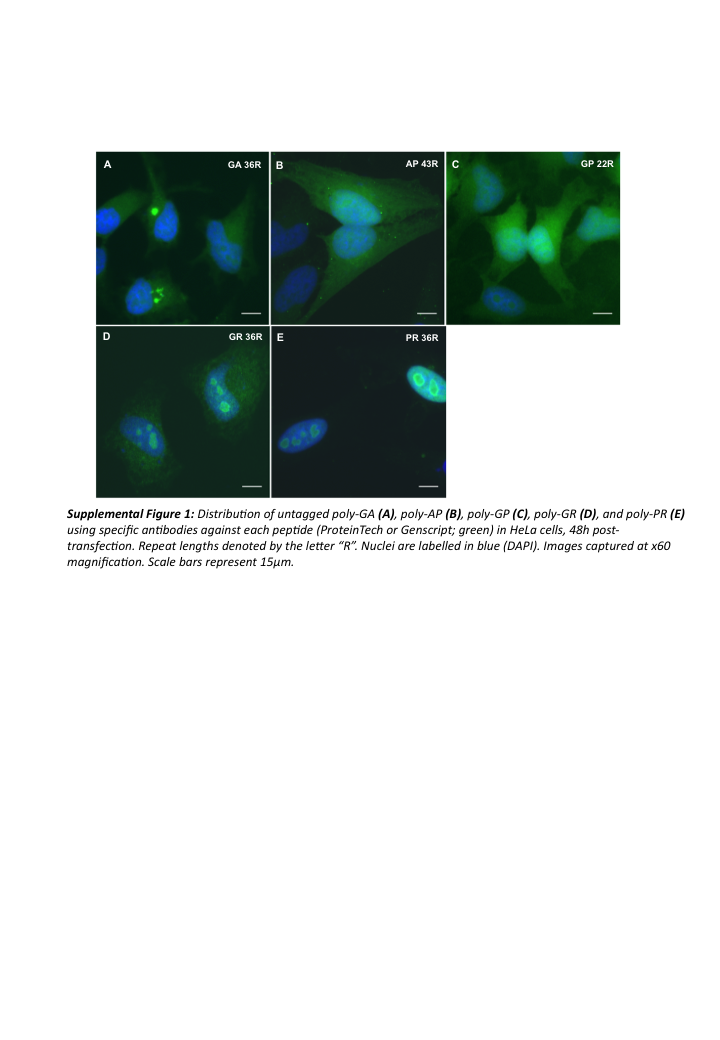


Supplemental Table 1

Supplemental Table 2

|  | GFP | GA36 | GA510 | GA1020 |
| --- | --- | --- | --- | --- |
|  |  | **Inclusion** | **Inclusion** | **Inclusion** |
| Holding current at -60 mV (pA) | -19.6 ± 3.8 *(11)* | -12.6 *(2)* | -17.7 ± 7.7 *(6)* | -19.0 ± 13.9 *(5)* |
| Input resistance (GΩ) | 2.4 ± 0. 3*(11)* | 2.2 *(2)* | 1.9 ± 0.2 *(6)* | 1.5± 0.4 *(5)* |
| Time course (ms) | 26.3 ± 3.9 *(11)* | 37.8 *(1)* | 16.9 ± 3.5 *(5)* | 18.1 *(2)* |
| Action potential properties |  |  |  |  |
| Amplitude (mV) | 60.6 ± 3.3 *(11)* | 40 *(1)** | 45.5 ± 5.5 *(3)** | 53.9 *(2)* |
| Threshold (mV) | -44.0 ± 1.5 *(11)* | -35.2 *(1)** | -38.4 ± 4.2 *(3)* | -42.8 *(2)* |
| Overshoot (mV) | 16.6 ± 3.5 *(11)* | 4.4 *(1)* | 6.6 ± 2.2 *(3)* | 11.2 *(2)* |
| 1⁄2 width (ms) | 7.2 ± 0.8 *(11)* | 14.0 *(1)** | 14.2 ± 2.6 *(3)*** | 5.3 *(2)* |
| Peak current at 30 mV (pA/pF) | 73.2 ± 8.6 *(11)* | 52.8 *(2)* | 33.0 ± 5.8 *(6)*** | 76.9 ± 13.9 *(5)* |

|  | GFP | AP43 | AP512 | AP1024 |
| --- | --- | --- | --- | --- |
| Holding current at -60 mV (pA) | -19.6 ± 3.8 *(11)* | -22.3 ± 6.6 *(5)* | -19.1 ± 4.7 *(6)* | -13.6 ± 4.0 (5) |
| Input resistance (GΩ) | 2.4 ± 0. 3*(11)* | 2.4 ± 0.5 *(5)* | 2.2 ± 0.3 *(6)* | 2.4 ± 0.4 *(5)* |
| Time course (ms) | 26.3 ± 3.9 *(11)* | 24.3 ± 3.7 *(5)* | 19.8 ± 1.9 *(6)* | 30.8 ± 5.6 *(5)* |
| Action potential (AP) properties |  |  |  |  |
| Amplitude (mV) | 60.6 ± 3.3 *(11)* | 71.4 ± 8.7 *(5)* | 54.9 ± 7.8 *(6)* | 53.4 ± 3.8 *(5)* |
| Threshold (mV) | -44.0 ± 1.5 *(11)* | -45.7 ± 1.0 *(5)* | -44.8 ± 1.7 *(6)* | -48.1 ± 1.4 *(5)* |
| Overshoot (mV) | 16.6 ± 3.5 *(11)* | 24.5 ± 8.5 *(5)* | 9.8 ± 7.0 *(6)* | 8.3 ± 2.9 *(5)* |
| 1⁄2 width (ms) | 7.2 ± 0.8 *(11)* | 6.4 ± 0.8 *(5)* | 7.8 ± 1.5 *(6)* | 7.9 ± 1.5 *(5)* |
| Peak current at 30 mV (pA/pF) | 73.2 ± 8.6 *(11)* | 71.6 ± 7.2 *(5)* | 81.0 ± 11.4 *(6)* | 65.7 ± 15.8 *(5)* |

Supplemental Table 3

|  | GFP | GR36 | GR142 | GR284 |
| --- | --- | --- | --- | --- |
| Holding current at -60 mV (pA) | -19.6 ± 3.8 *(11)* | -15.7 ± 3.1 *(5)* | -14.7 ± 7.1 *(6)* | -19.0 ± 1.0 *(4)* |
| Input resistance (GΩ) | 2.4 ± 0. 3*(11)* | 1.4 ± 0.4 *(5)* | 3.1 ± 0.6 *(6)* | 3.2 ± 1.3 *(4)* |
| Time course (ms) | 26.3 ± 3.9 *(11)* | 26.1 ± 4.2 *(5)* | 25.1 ± 4.0 *(5)* | 25.1 ± 7.8 *(4)* |
| Action potential (AP) properties |  |  |  |  |
| Amplitude (mV) | 60.6 ± 3.3 *(11)* | 55.4 ± 5.0 *(5)* | 55.5 ± 4.1 *(5)* | 47.7 ± 6.1 *(3)* |
| Threshold (mV) | -44.0 ± 1.5 *(11)* | -42.0 ± 0.7 *(5)* | -40.4 ± 0.7 *(5)* | -39.8 ± 3.2 *(3)* |
| Overshoot (mV) | 16.6 ± 3.5 *(11)* | 12.7 ± 4.5 *(5)* | 15.1 ± 3.7 *(5)* | 8.2 ± 4.6 *(3)* |
| 1⁄2 width (ms) | 7.2 ± 0.8 *(11)* | 7.4 ± 1.8 *(5)* | 7.0 ± 0.7 *(5)* | 10.3 ± 1.5 *(3)* |
| Peak current at 30 mV (pA/pF) | 73.2 ± 8.6 *(11)* | 99.0 ± 14.1 *(5)* | 59.3 ± 7.1 *(6)* | 26.0 ± 8.3 *(4)*** |

Supplemental Table 4

|  | GFP | PR36 | PR220 | PR550 |
| --- | --- | --- | --- | --- |
|  |  | GFP nucleus and nucleoli | GFP nucleus and nucleoli | GFP nucleus and nucleoli |
| Holding current at -60 mV (pA) | -19.6 ± 3.8 *(11)* | -34.8 ± 8.0 *(12)** | -37.0 ± 19.2 *(8)* | -31.3 ± 15.1 *(4)** |
| Input resistance (GΩ) | 2.4 ± 0. 3*(11)* | 1.4 ± 0.3 *(12)** | 1.5 ± 0.3 *(8)* | 2.1 ± 0.6 *(4)* |
| Time course (ms) | 26.3 ± 3.9 *(11)* | 22.0 ± 3.8 *(12)* | 19.8 ± 3.0 *(8)* | 27.1 ± 5.9 *(4)* |
| Action potential (AP) properties |  |  |  |  |
| Amplitude (mV) | 60.6 ± 3.3 *(11)* | 42.8 ± 5.2 *(10)** | 48.5± 3.2 *(5)** | 54.7 ± 1.4 *(4)* |
| Threshold (mV) | -44.0 ± 1.5 *(11)* | -39.2 ± 1.1 *(10)** | -42.8 ± 3.0 *(5)* | -44.4 ± 1.1 *(4)* |
| Overshoot (mV) | 16.6 ± 3.5 *(11)* | 3.4 ± 4.7 *(10)** | 6.7 ± 2.1 *(5)* | 10.6 ± 0.9 *(4)* |
| 1⁄2 width (ms) | 7.2 ± 0.8 *(11)* | 7.1 ± 0.8 *(10)* | 7.8 ± 1.1 *(5)* | 6.3 ± 0.5 *(4)* |
| Peak current at 30 mV (pA/pF) | 73.2 ± 8.6 *(11)* | 71.9 ± 6.7 *(12)* | 64.3 ± 6.2 *(8)* | 81.8 ± 6.6 *(4)* |
